# Solvent-Assisted Paper Spray Ionization Mass Spectrometry (SAPSI-MS) for the Analysis of Biomolecules and Biofluids

**DOI:** 10.1038/s41598-019-45358-x

**Published:** 2019-07-16

**Authors:** Nicoló Riboni, Alessandro Quaranta, Hitesh V. Motwani, Nicklas Österlund, Astrid Gräslund, Federica Bianchi, Leopold L. Ilag

**Affiliations:** 1 Department of Environmental Science and Analytical Chemistry, Stockholm University, Stockholm, SE Sweden; 2 Department of Biochemistry and Biophysics, Stockholm University, Stockholm, SE Sweden; 3 Department of Chemistry, Life Sciences, and Environmental Sustainability, University of Parma, Parma, IT Italy

**Keywords:** Mass spectrometry, Bioanalytical chemistry

## Abstract

Paper Spray Ionization (PSI) is commonly applied for the analysis of small molecules, including drugs, metabolites, and pesticides in biological fluids, due to its high versatility, simplicity, and low costs. In this study, a new setup called Solvent Assisted Paper Spray Ionization (SAPSI), able to increase data acquisition time, signal stability, and repeatability, is proposed to overcome common PSI drawbacks. The setup relies on an integrated solution to provide ionization potential and constant solvent flow to the paper tip. Specifically, the ion source was connected to the instrument fluidics along with the voltage supply systems, ensuring a close control over the ionization conditions. SAPSI was successfully applied for the analysis of different classes of biomolecules: amyloidogenic peptides, proteins, and N-glycans. The prolonged analysis time allowed real-time monitoring of processes taking places on the paper tip, such as amyloid peptides aggregation and disaggregation phenomena. The enhanced signal stability allowed to discriminate protein species characterized by different post translational modifications and adducts with electrophilic compounds, both in aqueous solutions and in biofluids, such as serum and cerebrospinal fluid, without any sample pretreatment. In the next future, application to clinical relevant modifications, could lead to the development of quick and cost-effective diagnostic tools.

## Introduction

Ambient mass spectrometry (AMS) has generated much interest in the last 15 years, given the possibility of analyzing target species at trace levels in complex matrices, requiring minor or no sample pretreatment^1–4^. AMS includes ionization techniques carried out under ambient and open-air conditions, allowing rapid, real-time, *in-situ* analysis, usually coupled to high-resolution mass spectrometry (HRMS)^5^. The consequent reduction of the analysis time enables the development of fast and reliable screening methods with possibility of quantitation^6^.

Among AMS techniques, paper spray ionization (PSI) has become popular due to its simplicity, versatility, sensitivity, and cost-effectiveness^7,8^. The sample is spotted on a triangular paper tip held in position by a stainless steel clip, and allowed to dry. Subsequently, few microliters of a suitable solvent are applied on the tip, and a potential (usually in the kV range) is applied to the clip, leading to the formation of a Taylor cone towards the MS inlet, as in electrospray ionization, resulting in the production of multi-charged ions^7–9^. Several parameters affect the ionization efficiency, including paper type and shape, solvent, and sample-to-paper ratio^8,9^. Paper thickness, mechanical resistance, surface chemistry, and fiber morphology have crucial influence on PSI performances^10^. Therefore, several functionalized papers have been developed for the analysis of specific targets^1^.

Since its development in 2010^7^, PSI has been successfully applied for the analysis of drugs and metabolites in biofluids^9,11^, forensics^12^, and food safety^13^. One of the major drawbacks of this technique is the short data acquisition time, due to the quick consumption of the ionization solvent^8^. Several modifications to the conventional PSI setup, including spotting automation, integration in cartridges, and direct tissue spray, have been proposed^1,3,11,14^. Different modifications dealing with solvent supply have been previously implemented, all requiring the use of external devices both for solvent supply and voltage delivery. Moreover, they were all targeted towards the detection of small molecules^15–18^, or limited to the exploration of ionization mechanisms in PSI^19–21^. In this study, an innovative setup integrating the PSI support with the power supply and the fluidics system of a Waters Synapt G2S nano-ESI source was developed. The proposed solution allows for the simultaneous and direct control of the applied voltage, solvent flow rate, and composition, extending the analysis time up to one hour. Under these conditions, real time monitoring of processes and reactions occurring on the paper can be easily performed. In addition, signal stabilization over time could increase the sensitivity and the resolution of the technique, extending the applicability of PSI towards the detection of large biomolecules, which has so far proven to be extremely challenging. To our knowledge, only few studies on intact standard proteins and complexes (angiotensin II, apolipoprotein C1, cytochrome c, concavalin A, hemoglobin, lysozyme, myoglobin, and transthyretin) based on the use of PSI have been reported^14,22,23^.

To test the possibilities opened by this setup, different systems were analysed by focusing on three classes of biomolecules: amyloid-β (1–40) (Aβ) peptide and its oligomeric forms, soluble intact proteins (mass range of 15–80 kDa) in the denatured form, and N-glycans. Aβ peptides are 39–42 residue long peptides that are intimately linked to Alzheimer´s disease (AD), as they aggregate to form so-called senile plaques found in AD patients brain. This phenomenon is at the core of the Amyloid Cascade Hypothesis^24^ and understanding its regulating factors could help to disclose AD-related mechanisms. Aggregation and disaggregation phenomena regarding Aβ peptides were investigated by monitoring the presence and the evolution of the oligomeric states in real time. The early oligomeric stages have previously been studied using nano-ESI, however problems with clogged electrospray needles, due to formation of large aggregates, have been reported^25^.

The analysis of intact non-covalent protein complexes in native form has been performed on classic PSI^23^. In the present study, we focused on the identification of post-translational modifications (PTMs) and covalent adducts with small molecules. Four different proteins were analyzed, namely human serum albumin (HSA), human hemoglobin (Hb), human transferrin (TFN), and bovine superoxide dismutase (SOD). Glycosylation was characterized on standard TFN both on the intact protein and on released acidic N-glycans. Neutral N-glycans were also analyzed after release from α-1-antitrypsin (AAT) and enzymatic cleavage of sialic acids. The detection of changes in the glycosylation pattern of proteins and in the relative abundance of specific protein species could be an intriguing field of application of PSI since these modifications are related to a large number of disorder^26–28^. Analysis of biofluids such as serum and cerebrospinal fluid (CSF) was performed without including any pretreatment. The possibility of analyzing stable protein adducts can be a valuable tool to assess exposure to potentially mutagenic molecules^29^ and could be used to build reliable and fast screening methods which would require only few microliters of blood.

## Results and Discussion

**Figure 1:**
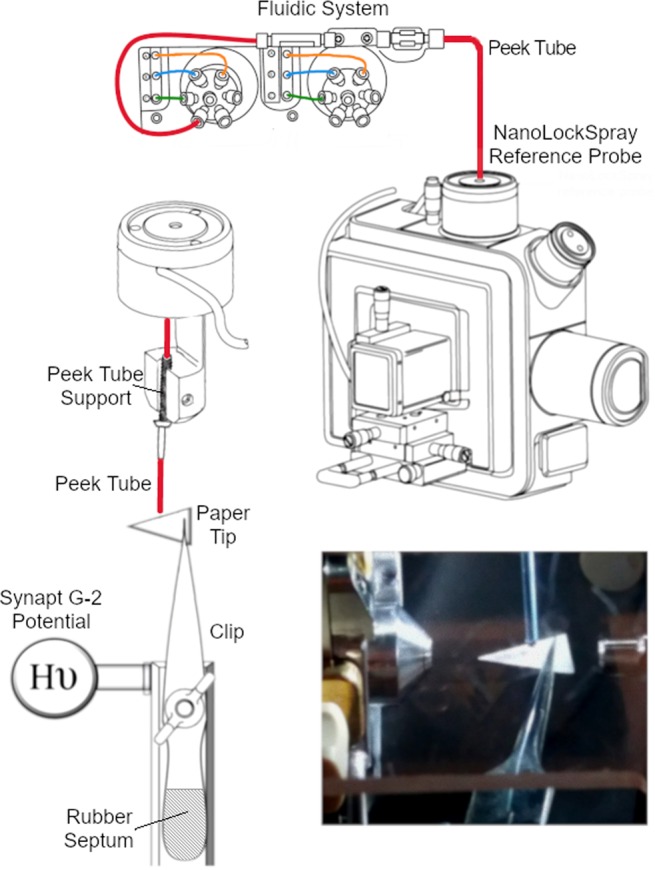
SAPSI ion source after modification of the Waters Synapt nano-ESI source, connected to the instrument´s fluidic system (adapted from Synapt Mass Spectrometry System Operator’s Guide with permission from Waters Sweden).

The Waters Synapt nano-ESI source was customized to host an integrated support for PSI and to include an integrated solvent supply system. The support for the PSI clip replaced the commercial nano-ESI needle holder and was directly connected to the power supply of the instrument, thus ensuring a reproducible, tunable, and verifiable voltage. Solvent supply to the paper was provided by modifying the NanoLockSpray reference probe to introduce a support for peek tubes and removing the existing capillary holder. The peek tubing was then integrated in the Synapt fluidics system allowing a precise control over the flow rate and the solvent composition via MassLynx software. The setup is illustrated in Fig. 1.

Different commercially available clips with varying shapes were tested, considering their ability to deliver the potential to the paper tip. The 18/10 stainless steel clip displayed in Fig. 1 (Bochem, Weilburg, DE), characterized by a very sharp edge, was able to deliver the applied potential without any measurable difference from the input value. Four types of commercially available paper were tested with regard to the ability of facilitating the ionization of the analytes: filter paper (Sigma-Aldrich, St.Louis, MO, USA), chromatographic paper grade 1, chromatographic paper 3MM CHR, and polydivinylidene fluoride (PVDF) membrane (GE Healtcare, Chicago, IL, USA). PVDF proved not to be suitable for the detection of compounds in polar solvents, due to the surface´s hydrophobicity, which prevented paper impregnation. Moreover, this non-conductive material did not present sufficient mechanical resistance to both the sample spotting and the solvent flow, resulting in bending and misalignment to the MS inlet. Chromatographic paper grade 1 was preferred over the 3MM because its reduced thickness enhanced performances in PSI MS^10^. Filter paper showed PSI-compatible mechanical properties but resulted in higher background noise and less reproducible ionization.

Different solvent mixtures were tested as reported in Table S3. The performance of PSI ionization depend on several solvent parameters, such as boiling point, viscosity, interaction with paper, surface tension and dielectric constant (ε)^8^. Solvents displaying low surface tension values facilitate the formation of the electrospray from the paper tip, whereas high ε increases the conductivity of the system. Equivolumic mixtures of IPA and acetonitrile (ACN) with water provided the best results for all the analyzed classes of compounds. This could be attributed to their characteristic low surface tension (24 and 33 mN m^−1^ respectively, at 25 °C) and medium-high values of ε (43 and 55 respectively, at 25 °C). The addition of formic acid within 0.1–1% v/v range was required for ionization.

### Real-time monitoring of Aβ peptides and its aggregates

Real time monitoring was applied to the study of aggregation and disaggregation phenomena of Aβ (1–40). The analysis of concentrated solutions of monomer standard was performed to assess the durability of the analysis and evaluate different solvent mixtures. High water content resulted in scarce ionization, high noise, aggregation, and difficulties in declustering. Moreover, considering that amyloid fibrillar aggregates are water insoluble, aqueous solvent mixtures could enhance the presence of artifact aggregates or on-paper irreversible adhesion/adsorption. Thus, the addition of apolar solvents to the elution phase was tested, resulting in high intensity signals lasting for at least 90 minutes, using flow rates up to 5 µL/min.

To assess the possibility of observing larger aggregate species, in-solution incubation of concentrated standard monomer was performed and analyzed. Since large oligomers are highly apolar, suitable solvent mixtures were tested to enhance ionization, namely methanol:toluene 1:1 with HCOOH 1% v/v and different H_2_O:ACN and H_2_O:IPA combinations. The best results were obtained by using H_2_O:IPA 1:1, HCOOH 1% v/v. As reported in a previous study^30^ IPA, in addition to providing the above described ionization advantages, could promote desorption from the paper and the stripping of monomeric units from the aggregates.

**Figure 2:**
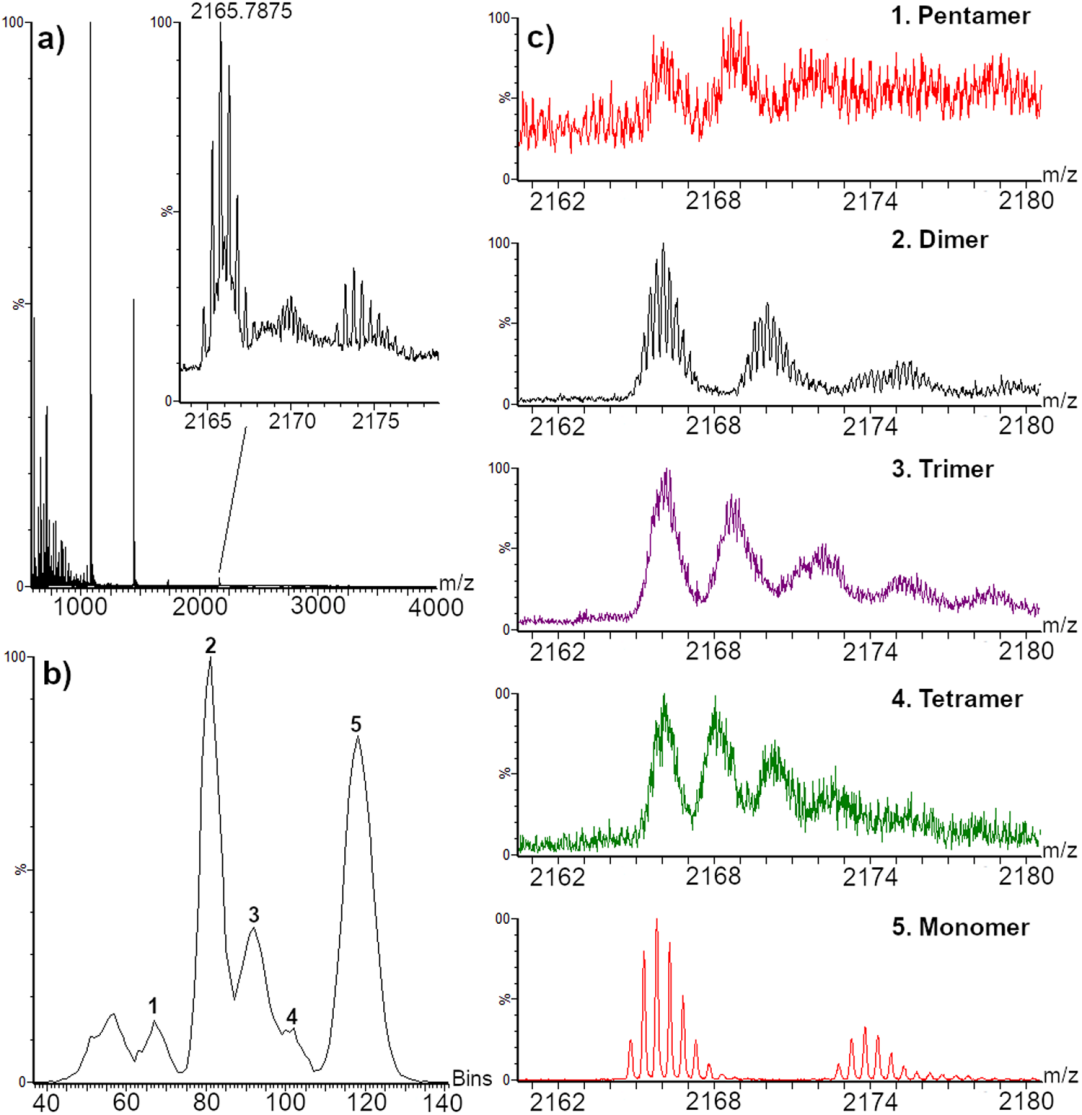
MS and IMS spectra of aggregated Aβ peptide from 0 to 4 minutes of analysis a) MS spectrum, magnified around m/z 2165. b) IMS spectrum extracted from m/z 2165. c, 1 to 5) MS spectra extracted according to the different drift times.

The differentiation of oligomeric states required the coupling with ion mobility spectrometry (IMS), since ions generated by different oligomers can share the same m/z ratio. IMS allows resolving overlapped MS signals based on their collisional cross section (CCS) and charge^31^. In Fig. 2, the MS and IMS spectra of m/z 2165.95 are shown. Five oligomers could be identified based on their different drift time and isotopic pattern. Interestingly the drift time order for the oligomers differed from what was previously observed using nano-ESI, with trimer and tetramer being more elongated^32^. Differences in aggregation rate upon interactions between Aβ and different surfaces have been previously reported^33^. Our results would indicate that surfaces could greatly affect the aggregation pathway of the Aβ peptide already in the oligomeric stage. The presented methodology could therefore be used for systematic studies of interfacial processes.

Ions attributable to non-covalent oligomers ranging from dimers to octamers were identified. In Table S2, the ions observed for each oligomeric state are reported. The detection of species up to the hexamer proved to be comparable to more established techniques, such as nano-ESI^32^. Species correlated to the presence of heptamer and octamer were also observed, even though the identification was based on the presence of only one or two ions respectively.

Real time monitoring of the aggregation state was performed by acquiring spectra over one hour, divided in 4-minute intervals, and identifying the biggest specie present in each interval. As reported in Figure S1, it was possible to observe disaggregation leading to the presence of only monomeric and dimeric Aβ peptide after 20 minutes. Again, the disaggregation of large species and the sequential decrease in size could be explained by considering the amphiphilic nature of IPA, able to solubilize and scavenge Aβ units from bigger aggregates^30^.

### Analysis of intact proteins and modifications

HSA and TFN were used as model compounds to test the detection conditions in terms of source parameters and solvent composition for the analysis of intact proteins. These conditions were evaluated examining resolution between charge states, signal-to-noise ratio, repeatability of the deconvolution, and resolution of the deconvoluted peaks. The mixture H_2_O:IPA 1:1, HCOOH 1% v/v provided the best results. The effect of the solvent on the ionization of TFN is reported in Figure S2. By applying a constant flow of solvent, spray and evaporation were constantly compensated for by a fresh supply of mobile phase, keeping the composition constant and avoiding differential charging of the analytes over time. This resulted in narrower peaks for the respective charge states, thus improving the resolution. Moreover, the prolonged analysis time allowed summing a larger number of scans increasing the sensitivity. As a further advantage, it was possible to finely tune the position of the paper tip in relation to the cone in the beginning of each analysis while controlling in real time the signal response.

**Figure 3:**
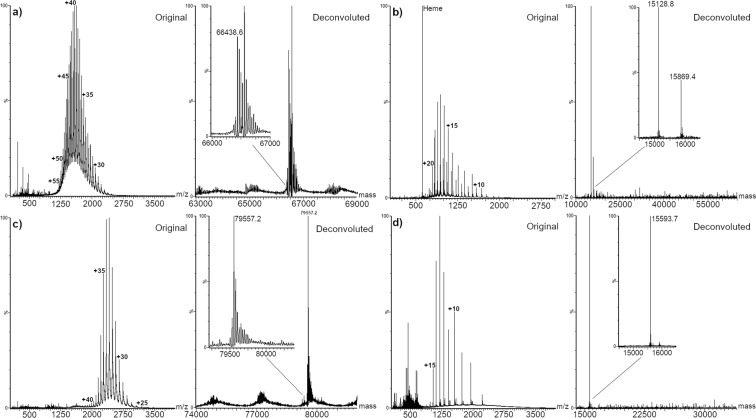
MS spectra and MaxEnt1 deconvolution of: (**a**) Human serum albumin, (**b**) Hemoglobin, (**c**) Transferrin, (**d**) Bovine superoxide dismutase.

Four standard proteins were analysed: HSA, TFN, Hb, and SOD (Fig. 3). HSA is the most abundant protein in serum, with concentration ranging between 30 and 50 mg/mL (450–750 µM)^28^. It is a monomeric multi-domain protein consisting of 585 amino acids with a molecular mass of 66.4 kDa. Among its 35 cysteine residues, 34 are involved in disulfide bridges, while the free Cys34 represents a pool of free thiol groups in serum, acting as scavenger antioxidant. In healthy adults, only a small percentage of Cys34 residues are oxidized to the sulfinic or sulfonic acid form^34,35^. Besides Cys34, other amino acids in HSA are prone to oxidation, i.e. methionine, histidine, arginine, and lysine. The increased content of carbonyl groups is considered a marker of oxidative stress derived from diseases such as liver failure, diabetes mellitus, sepsis, chronic renal failure, rheumatoid arthritis, or AD^35^. In addition to oxidation, other small compounds can bind Cys34, via disulfide bridge, like glutathione, homocysteine, or another cysteine^35^. Increased cysteinylation on Cys 34 has been related to chronic liver and kidney diseases, diabetes mellitus, and oxidative stress^28^. Glycation and glycosylation of HSA are considered as important parameters to be monitored as high levels of glycated proteins are often related to diabetes^35^.

Hb is the predominant protein in red blood cells and is responsible for transporting oxygen, carbon dioxide, and protons between the lungs and tissues. It is a tetramer weighing about 64.5 kDa, consisting of two α (15.75 kDa) and two non- α subunits (usually β, γ, or δ; 16.5 kDa). Common modifications in Hb are glycation and adduct formation with glutathione. Glycated Hb is a well-established parameter used in diabetes control, and it has been proposed as marker for atherosclerosis and other cardiovascular diseases^36^.

TFN is a non-heme iron-transport glycoprotein with two iron binding sites consisting of 679 amino acids, with a molecular weight of about 75 kDa (without considering glycosylation) and serum concentration ranging between 2 and 3 mg/mL (25–40 µM)^37^. Two N-glycosylation sites at Asn413 and Asn611 are mostly occupied by biantennary disialylated glycans. Alteration in the glycosylation pattern of TFN has been reported in several types of cancer, idiopathic normal pressure hydrocephalus, and neurodegenerative disorders^27,37^.

SODs are the main antioxidants against superoxides in mammals, consisting in three isoforms. The cytoplasmic Cu/ZnSOD (SOD1) is a homodimer of 32.5 kDa molecular weight which catalyzes the conversion of O2∙− to H_2_O_2_, which is finally decomposed to H_2_O. All the analyzed proteins were detected in the denatured form, as reported in Fig. 3.

SAPSI ionization of HSA resulted in charge states +27 to +57. The mass value obtained by deconvolution for the non-modified protein was 66438.6 ± 1.0 Da. Hb ionization under SAPSI conditions caused the disruption of the non-covalent complex with heme groups, as well as the reduction of the different subunits. Both subunits resulted in charge states +8 to +20, and the mass value obtained by deconvolution was 15128.8 ± 0.2 Da and 15869.4 ± 0.3 Da for the α and β subunits respectively (oxidized form of the β subunit was observed at 15902 Da). The base peak of the spectrum was assigned to the heme group (measured m/z 616.28, theoretical m/z 616.499) and confirmed by MS/MS analysis (Figure S3). Finally, ionization of TFN resulted in the charge states +25 to +43, providing a deconvoluted mass value of 79557.2 ± 1.2 Da, while SOD ionized in charge states +7 to +15, with a deconvoluted mass of 15593.7 ± 0.3 Da.

**Figure 4:**
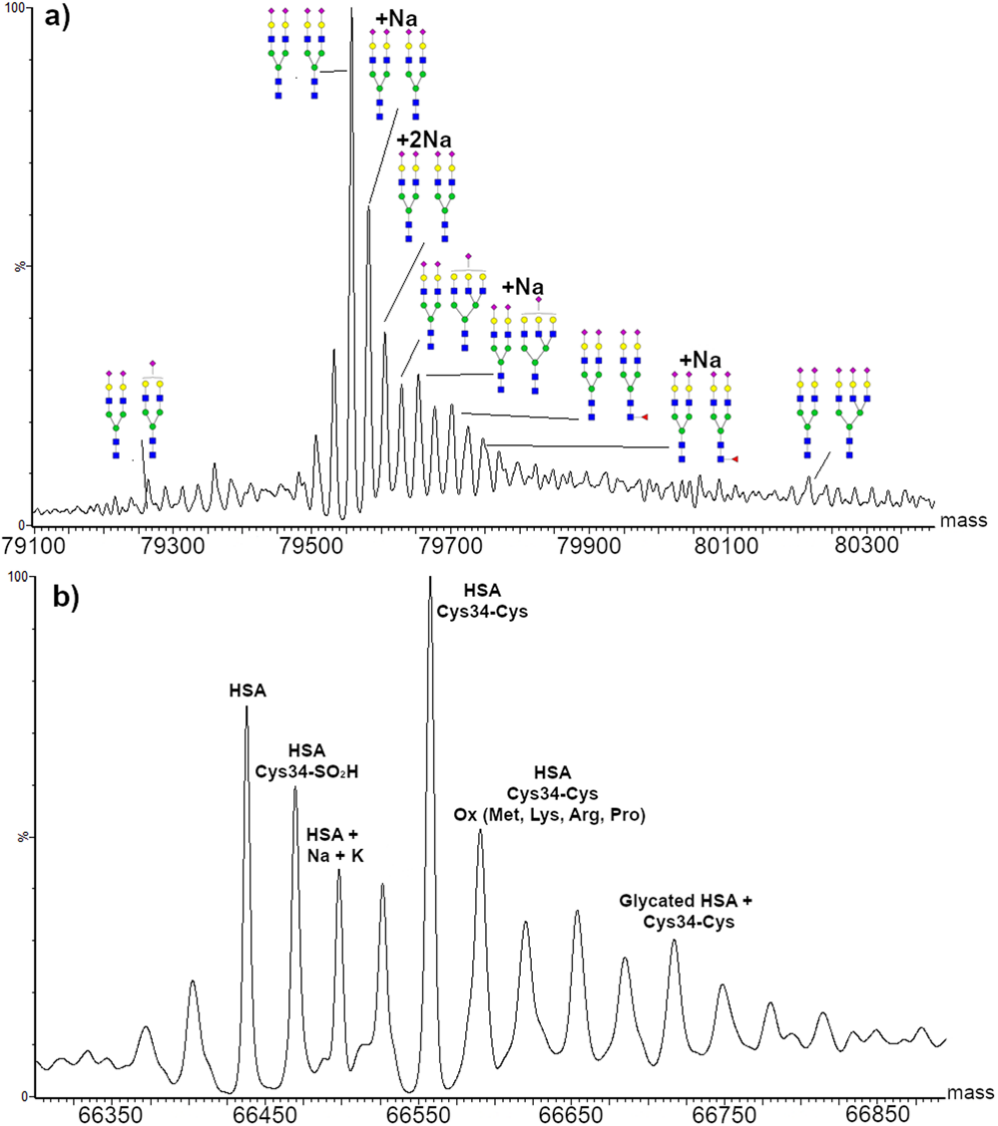
Identification of different transferrin and human serum albumin species. MaxEnt1 deconvolution of (**a**) standard human serum albumin with identification of different protein species and (**b**) standard transferrin with identification of different glycoforms.

The resolution obtained for the deconvoluted spectra allowed identifying different HSA and TFN species (Fig. 4). The base peak detected in HSA was assigned to the cysteinylated form (66557.9 ± 1.0 Da). Oxidation of the cysteinylated HSA and glycation were observed at 66590.1 ± 1.0 and 66717.0 ± 1.0 Da respectively. Modifications on non-cysteinylated HSA were also detected: oxidation of Cys34 to sulfinic acid (66469.6 ± 1.0 Da), and adduct with sodium and potassium cations (66498.2 ± 1.0 Da). The obtained results are in agreement with MS detection of oxidation and cysteinylation on intact HSA already reported in blood^38^ and in commercially available HSA standard^39^. The presence of intense peaks due to salt adducts is related to the presence of cations, mostly Na^+^ and K^+^, on untreated paper which can cluster with the proteins^23^.

The main glycoform identified in TFN consisted, as expected, of two biantennary disialylated glycans H5N4S2. All the identified glycoforms maintained one H5N4S2 glycan, whereas the second differed, having various sialylated and fucosylated bi- and tri-antennary species. Intense signals were assigned to TFN bearing two H5N4S2 glycans adducted with one or two sodium ions: this coordination is extremely common in glycans, as the hydroxyl groups in the sugar residues are known to easily coordinate alkali cations. Since cellulose is known to have high affinity for alkali cations, this phenomenon was probably enhanced when using untreated paper.

### Analysis of N-glycans

Analysis of glycosylation on intact proteins provides information about the different glycoforms, but cannot discriminate between positional isomers, characterized by heterogeneity in position of fucosylation, bisecting N-Acetyl glucosamine, and position and linkage of sialic acids. Changes in these features have been related to several types of cancer and neurological diseases, thus they can be used as biomarkers^26,27^.

Analysis of glycosylation was performed on standard H5N4S2 glycan and enzymatically released N-glycans, both with and without sialic acids. Standard H5N4S2 was used to tune source parameters and solvent composition for this class of compounds that, to our knowledge, has never been analyzed by PSI. The best performances were obtained by using H_2_O:ACN 1:1, HCOOH 0.1% v/v. The lower amount of acid is explained considering the high affinity of glycans for alkaline cations: higher percentages of acid in the solvent resulted in the enhancement of triply charged ions that complicated the spectra and caused a sensitivity drop due to the splitting of the signal. Furthermore, the lower amount of acid allows reducing the ionization of potentially interfering species. Since the proposed setup extended the analysis time, MS and MS/MS analyses could be easily performed in the same run.

**Figure 5:**
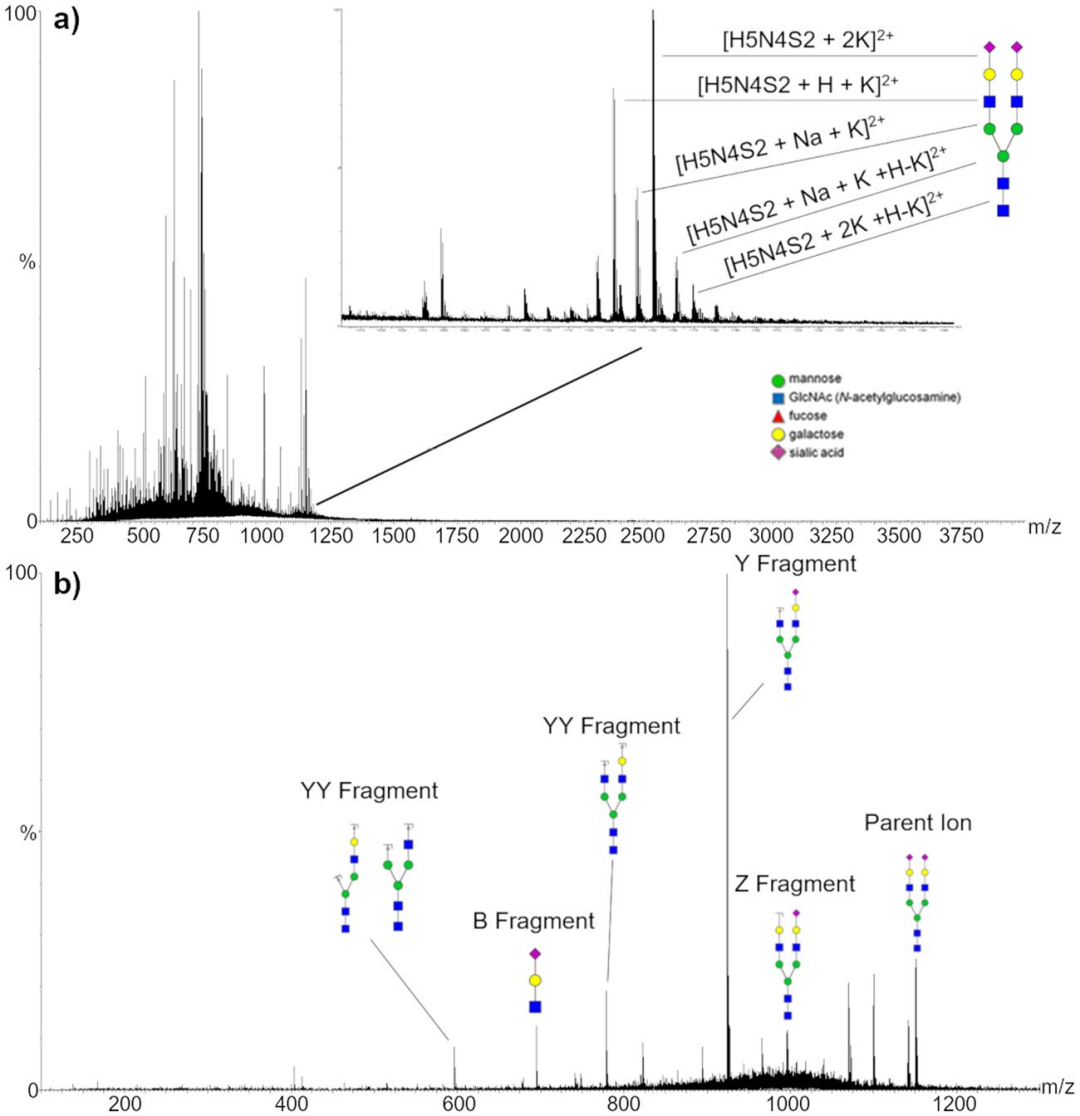
MS spectrum from the analysis of standard H5N4S2 glycan (**a**) and MS/MS fragmentation of m/z 1150.3110 and identification of the generated fragments (**b**).

Analysis of H5N4S2 standard glycan resulted in a combination of doubly-charged adducts with sodium and potassium cations. The most intense signal attributable to the analyzed glycan was at m/z 1150.3110 corresponding to [H5N4S2 + 2 K]^2+^. Five fragments were identified by MS/MS on this peak providing full structural elucidation. MS and MS/MS spectra are reported in Fig. 5.

Acidic glycans were released from TFN and the results were compared to what already observed on the intact protein. As displayed in Figure S4, only double-charged adducts of H5N4S2 with sodium and potassium could be identified. Deglycosylated TFN was still present in the sample, but ionized poorly due to the low amount of acid and to the ACN-based solvent.

As already mentioned, TFN has two glycosylation sites: the exposed Asn432, presenting mostly H5N4S2 glycan, and the Asn630, buried in the quaternary structure, showing more heterogeneity^37^. The absence of glycans other than H5N4S2 could be due to their low occurrence on TFN and to the protein not being denatured before the enzymatic treatment. Structural analysis by MS/MS was performed on [H5N4S2 + 2 K]^2+^ resulting in the identification of six fragments matching the standard fragments.

For the analysis of neutral glycans, the release was performed together with enzymatic desialylation from AAT. The choice of this protein was due to the larger variety of core structure expressed if compared with TFN. In addition, alteration in the glycosylation pattern of AAT in CSF has been related to AD^40^, whereas in serum to chronic and acute inflammatory processes^41^. Four species could be identified corresponding to H5N4 (split in four different forms), H5N4F, H6N5, and H6N5F, detected as alkali adducts (Figure S5). Fragmentation was performed on the most intense signal related to H5N4 [H5N4 + H + K]^2+^, yielding six fragments and full structural elucidation (Figure S6).

### Biofluids

**Figure 6:**
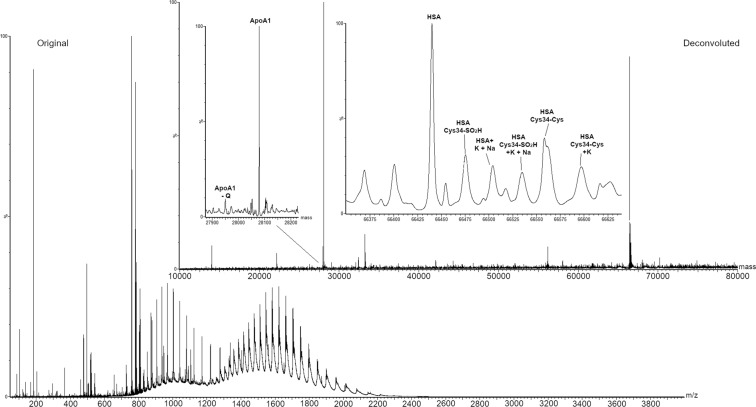
MS spectrum of diluted human serum and related MaxEnt1 deconvolution. Magnifications highlight apolipoprotein A1 and human serum albumin regions, with identification of detected protein species.

Not requiring any sample pretreatment, PSI is commonly used for the analysis of biofluids such as blood, serum, urine, and saliva. In this work, human serum and CSF were tested as biological matrices after simple dilution, with focus on the identification of intact biomolecules, specifically lipids and proteins. Figures 6 and S7, 8 report the results obtained for human serum and CSF respectively.

Serum analysis revealed the presence of two charge-series envelopes, together with an intense group of signals around m/z 760. The envelope centered around m/z 1500–1600 matched the signals obtained for HSA in standard solution. HSA is the major component of the serum protein pool, accounting for more than 50% of the total^28^. The lower m/z envelope centered on m/z 1000 also presented very intense peaks. To identify the unknown protein and detect the presence of minor components, a wide-range deconvolution between 10 and 80 kDa was performed within the region between m/z 800 and 3000. As displayed in Fig. 6, an intense peak with a mass of 28079.0 ± 0.5 Da was calculated together with the HSA peak. Exact mass database search matched with Apolipoprotein A1 (ApoA1) with an accuracy of 14 ppm (0.4 Da on 28 kDa) on the mass calculated from the amino acid sequence. ApoA1 is reported to undergo glycation and present a truncated variant deriving from the loss of a Q at the C-terminus (27950.5 ± 0.5 Da)^42^. Even though the glycated isoform could not be observed, the truncated form was present and matched the theoretical value with an accuracy of 3.5 ppm (0.1 Da on 27.9 kDa). It is reported that ApoA1 glycation and truncation were related to atherosclerosis and impairment in its anti-inflammatory action^42^. The reference concentration value for ApoA1 in serum is of 1.4 mg/mL (50 µM). Even though this value is more than 10 times lower if compared to HSA average values, ApoA1 showed a major density of charge than HSA, explaining the comparable intensity of the two envelopes.

Six HSA species were identified, the base peak corresponding to the unmodified protein, followed in intensity by the oxidized Cys34 and the cysteinylated Cys34 forms. Sodium and potassium adducted forms were also present, having lower intensities. Compared to the standard, less cysteinylated and oxidized forms were detected. The absence of glycation in both proteins can be a consequence of the samples´ conditions, as glycation is not enzyme-driven and depends on glucose levels in blood.

The signals in the region between m/z 758 and 782 were initially attributed to lipids. The obtained mass values and the high ionization efficiency were compatible with lipids belonging to the phosphatidylcholine (PC) class. These lipids, commonly present in blood^43^, present a permanent charge on the quaternary nitrogen that explains the high ionization yield. The identification was confirmed by comparison with standard PC 16:0–18:1, bearing palmitic and oleic acid (molecular weight 760.568 Da, Figure S9). The fragmentation pattern of the standard matched perfectly the one obtained in serum (Figures S10, S11). The identification of PC 16:0–18:1 and MS/MS fragmentation of m/z 758.576 (Figure S12) allowed the assignment of the peak as PC 16:0–18:2, whereas peaks at m/z 780.554 and, 782.568, and 784.584 were assigned to the sodiated adducts. Doubly sodiated adducts were observed at m/z 802.537, 804.554, and 806.573 Da (Figure S9). Alterations in the metabolism and concentration of PCs have been related to AD and several types of cancer^43^.

Analysis of CSF revealed the same components as the ones identified in serum, as expected with lower intensity. Both ApoA1 and HSA were detected and several modifications were identified, with the prevalence of oxidized, cysteinylated, and glycated species. Regarding HSA, the base peak corresponded to the cysteinylated form and singly and doubly glycated HSA peaks were also observed at 66728 ± 1.0 and 66891 ± 1.0 Da. Oxidized forms of the first two were present both as unmodified and adducted with alkali cations, whereas unmodified HSA accounted for only 25% of the base peak in the deconvoluted spectrum. ApoA1 exhibited a very intense peak at 28128 ± 0.5 Da, consistent with oxidation of a Met residue. The glycated form of oxidized ApoA1 could also be observed at 28290 ± 0.5 Da, whereas unmodified ApoA1 and its sodium adduct were detected at 28079 ± 0.5 and 28102 ± 0.5 Da respectively (Figure S8). The high incidence of oxidation in the two proteins could be related to oxidative stress while glycation, as mentioned above, could be a consequence of high glucose levels in the individual.

### Covalent adduct detection of intact proteins in standard solution and in human serum

The possibility of discriminating among different HSA adducts from small molecules (<100 Da) in blood by using SAPSI, would demonstrate the potential of this technique for *in vivo* studies, concerning exposure and metabolism of electrophilic mutagenic compounds. For this purpose, acrylamide (AA), a small electrophile (molecular weight 71 Da), was used as a model alkylating agent. AA is generated when food is cooked at high temperatures^44^ and is classified as group 2 A by IARC^45^. In adductomics, HSA and Hb are commonly used as they are very abundant in blood, have a long half-life, and form stable adducts^29,34^.

Alkylation was first performed in aqueous solutions of standard HSA or Hb (physiological concentration), at two different AA concentration levels, i.e. 30 µM and 0.3 µM. MS spectra and deconvolution results are shown in Figures S14 and S15. No adduct could be detected at the lower AA level for both proteins, whereas mono and double AA adducts on HSA were observed at the higher level. Mono AA adducts were also observed in both α- and β-chains in Hb.

**Figure 7:**
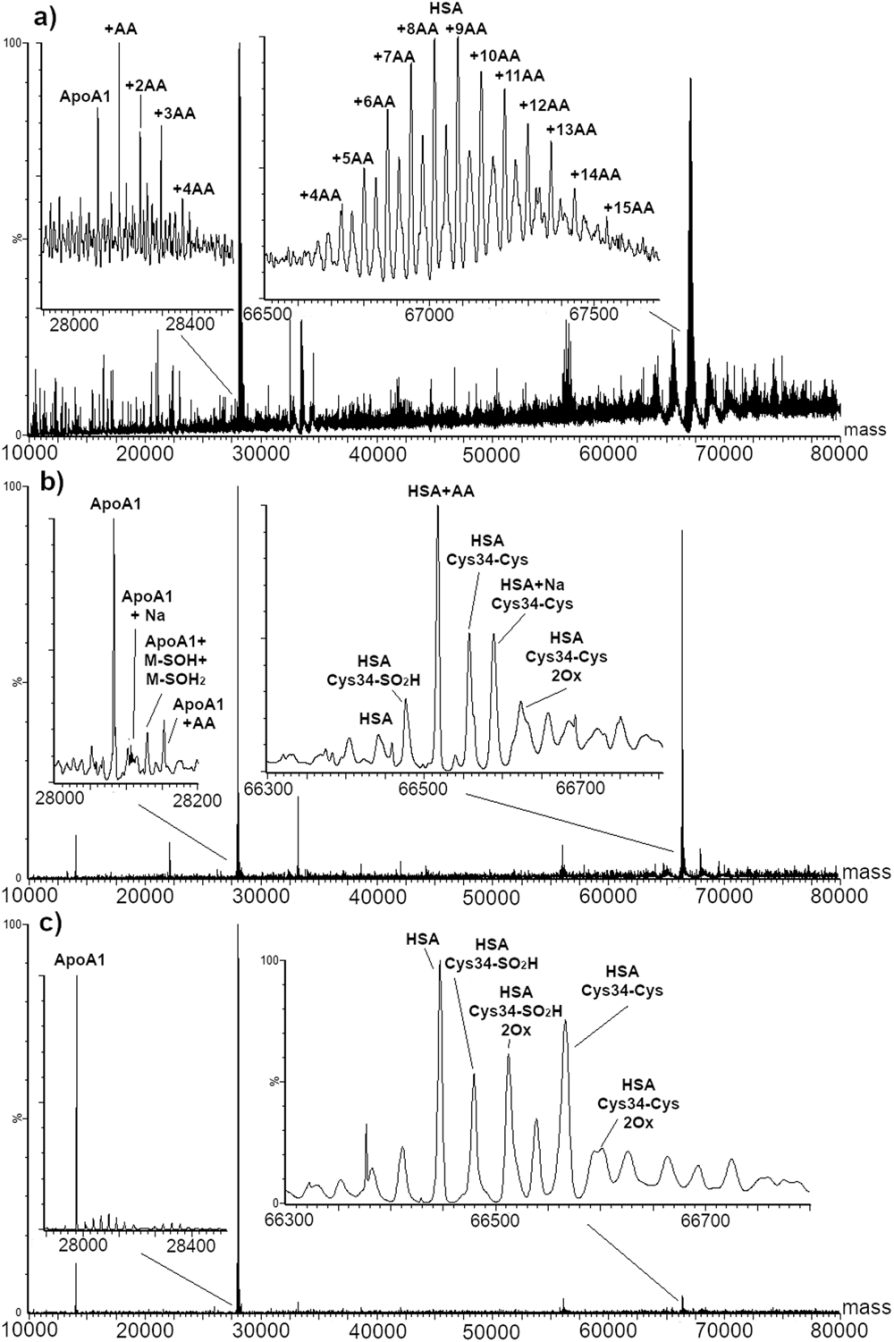
MaxEnt1 deconvolution of serum incubated with acrylamide at: 30 µM (**a**), 3 µM (**b**), and 0.3 µM (**c**). Magnifications show apolipoprotein A1 and human serum albumin regions with the identified species.

Serum was incubated with three AA concentration levels, from 0.3 to 30 µM. The results of the spectral deconvolution are displayed in Fig. 7.

Adducts to both HSA and ApoA1 were detected in serum after incubation with 3 and 30 µM AA. At 30 µM AA HSA was heavily alkylated: the first HSA peak detected was shifted by 284 Da from the unmodified mass value, corresponding to the HSA-4AA specie. Further adduct addition up to 18 AA units, with a maximum intensity for the HSA-9AA specie was observed. Simultaneously, the same trend could be observed for Cys34-SO_2_H HSA. Regarding ApoA1, the non-adducted form could still be detected, even though the most intense peak was related to the ApoA1-AA specie, and adduct species were present up to ApoA1-4AA.

Decreasing the concentration of AA in the incubated sample to 3 µM resulted in the identification of only mono-adducted HSA and ApoA1. These AA concentrations are comparable to those employed in animal studies for estimation of the internal dose of AA^46^. No adducted species could be observed at the lowest AA concentration level. Noticeably, alkylation in serum looked more efficient if compared to standard solution, despite the same molar ratio, incubation conditions, and pH. Future studies are required to address this issue and to increase the method sensitivity.

## Conclusions

To overcome common limitations of PSI related to solvent consumption, a new source design integrated with the power supply and fluidics system of a Waters Synapt G2S instrument was proposed. The presented setup, named SAPSI, was successfully applied for the analysis of biomolecules and biofluids. The coupling with IMS was used for the resolution of Aβ 1–40 peptide and its aggregates, allowing the investigation of aggregation and disaggregation phenomena over time directly on a surface by MS. The results highlighted the possibility of using SAPSI for the real-time monitoring of chemical and physical processes taking place on the paper tip, including surface effects that influence the structure and properties of biomolecules. This approach could be directed to study protein aggregation diseases such as AD, avoiding in-needle aggregation and clogging.

Application of SAPSI to the analysis of intact proteins allowed identifying different kinds of PTMs and adducts, as the resolution was sufficient to obtain baseline separation between forms having a mass difference of 15 Da. The technique was applied for the analysis of untreated biofluids, namely human serum and CSF. PTMs as glycosylation, oxidation, cysteinylation and glycation could be observed both in standard and in real samples. The characterization of these modifications could lead to the detection of biomarkers for several diseases. Protein adducts with exogenous electrophiles were also identified, both in aqueous solution and in serum, after incubation. The untargeted detection of adducts with high abundance proteins can be used as a tool to evaluate the exposure to toxic and carcinogenic compounds.

The low amounts of sample required and the long analysis time provided by SAPSI proved to be extremely beneficial for developing methods and performing analyses on scarcely available analytes or matrices. In the case of N-glycans, for which standards are usually very expensive, it was possible to tune the MS and MSMS conditions during a single run, while for typically limited matrices such as CSF, only a few microliters were necessary for a complete analysis.

The present results are a first step towards the development of fast, reliable, and cost effective screening methods able to provide a first indication of altered physiological states.

## Methods

### Human serum and CSF samples

Reference human serum samples were acquired from healthy donors through Karolinska Institutet, Sweden. Once received, the samples were aliquoted into microtubes and stored at −80 °C. CSF samples were obtained from the Centre for Neurodiagnostics biobank at the Uppsala University Hospital. Sample collection (2005-244 and Ö 48-2005) was approved by the Swedish Ethical Committee and all the experiments were performed in accordance with the associated guidelines and regulations. All the collected samples were obtained with the written informed consent of the individual donors.

### Instrumental setup

Source modification, parameter optimization, and all the experiments were performed using a Waters Synapt G2-S (Waters, Manchester, UK). After removing the nano-ESI needle holder, the source was modified by adding a custom-made clip holder, fitting the source support and connected to the Synapt power supply. Solvent supply was ensured by connecting the source to the instrument fluidics system using a peek tube (Fig. 1). Different peek tubing, with regard to inner diameter were tested considering wettability of the paper, solvent flow stability and viscosity. For all the tested solvent mixtures, peek having 0.10 mm inner diameter (i.d.) were used except for mixtures containing isopropanol (IPA) which, due to the higher backpressure, required peeks having 0.25 mm i.d. The paper tips were cut by using a homemade mold as isosceles triangles with a base of 6.0 mm and a height of 10.0 mm. Each tip was cut with the sharpest angle being 45 degrees, to suppress spray from the other corners^9^. The tips were positioned approximately 2–3 mm below the peek tube and 0.5 cm from the MS inlet. After positioning, 20 µL of sample solution were spotted onto the tip and the potential applied. The flow rate was set according to each solvent composition, ranging from 2 to 10 µL/min, ensuring consistent MS signal up to one hour. The source parameters were: cone voltage 120 V, source offset 80 V, cone gas 100 L/h, mass range 50–4000 m/z, scan time 1.0 second. Source temperature was set at 70–90 °C depending on the analyzed compounds, while the potential applied to the clip ranged from 3.5 to 4 kV. Detailed parameters used for the different applications are reported in Table S1.

### Amyloid peptide analysis

Aβ (1–40) stock solution (1 mg/mL) was prepared by dissolving the peptide in hexafluoroisopropanol, to dissolve eventual aggregates to monomeric form^47^. Samples were then prepared by drying an aliquot of the peptide stock under a stream of nitrogen, forming a thin peptide film. The peptide film was resolved in 5% ammonium hydroxide solution and sonicated on ice for 1 minute. The sample was then diluted to 0.25 mg/mL in aqueous 50 mM ammonium acetate solution and spotted on the paper tip. The fluidics was started immediately to avoid drying-induced aggregation.

Aggregated samples were obtained by incubation of samples (0.25 mg/mL Aβ in 50 mM ammonium acetate solution) for one hour at 37 °C under agitated conditions (shaking at 750 rpm). Source temperature was set to 70 °C. IMS conditions were set as follows: IMS wave velocity 750 m/s, IMS wave height 25 V, Transfer cell Wave velocity 248 m/s, Transfer cell wave height 10 V, Trap gas 5 mL/min, Helium gas 100 mL/min, and IMS gas 80 mL/min. Data treatment was performed by MassLynx 4.1 and Driftscope 2.0.

#### N-Glycan analysis

Acidic glycans were enzymatically released from standard TFN by using the Peptide N-glycosidase F (PNGase F) kit for enzymatic release of N-linked glycans (New England Biolabs UK Ltd., Hitchin, UK) following the manufacturer’s instructions. After deglycosylation, the glycans were purified using porous graphitized carbon solid-phase extraction spin tips (Hypersep, 10–200 µL, Thermo Scientific, Waltham, MA, USA), in accordance with the manufacturer’s instructions. The release of neutral glycans from AAT was performed following the same procedure with the addition of 100 units of α-2-3,6,8 Neuraminidase (New England Biolabs UK Ltd., Hitchin, UK) to cleave the terminal sialic acids. Glycans were analyzed using a source temperature of 90 °C and trap collision energy for MS/MS in the 28–33 V range. Obtained glycan fragments were identified with the help of Glycoworkbench v2.1^48^. The following abbreviations were used in describing the composition of the identified glycans: H, Hexose; N, N-Acetyl hexosamine; S, Sialic acid; F, Fucose. Monosaccharides in the figures were represented following the SNFG (Symbol Nomenclature for Glycans) system^49^.

### Intact proteins, biofluids, and adducts analysis

Intact proteins standard solutions were prepared in water, 1% v/v HCOOH at the following concentrations: HSA 15 µM, TFN 12 µM, Hb 15 µM, and SOD 30 µM. All standards were purchased from Sigma-Aldrich (St.Louis, MO, USA). Human serum and CSF were spotted after dilution in water +1% v/v HCOOH (serum 1:30, CSF 1:3 v/v). The acquired spectra were deconvoluted by MaxEnt1 Deconvolution Software within the 20–120 kDa mass range, setting a resolution of 0.1 Da for the identification of proteins, The software was allowed to iterate until convergence, using a uniform Gaussian model (0.800 Da width at half height), with minimum intensity ratios of 33% for both left and right sides. Lipids were fragmented by MS/MS (CE 25–35 V) and identification was based on confront to a standard.

Adducts to standard proteins were obtained by incubating 50 µL of HSA and Hb solution (450 µM and 23 µM respectively) at physiological concentration in PBS (pH 7.4), with AA at three different concentration levels, namely 0.3 µM, 3 µM, and 30 µM. Incubation was performed overnight at 37 °C under shaking (750 rpm) and the resulting solutions were diluted 30 times before spotting. Similarly, adduct formation in serum was obtained by incubating 50 µL of serum with the same amounts of AA following the same procedure.

## Supplementary information

Supplementary information

## Data Availability

Data available on request.
