# Supplementary material for: Solvent-Assisted Paper Spray Ionization Mass Spectrometry (SAPSI-MS) for the Analysis of Biomolecules and Biofluids
Source: Sci Rep. 2019 Jul 16;9:10296. doi: 10.1038/s41598-019-45358-x (PMC6635430; doi:10.1038/s41598-019-45358-x)
Supplement: Supplementary file 1 — Supplementary information [file 41598_2019_45358_MOESM1_ESM.docx]

# Supporting information for:

# Solvent-Assisted Paper Spray Ionization Mass Spectrometry (SAPSI-MS) for the Analysis of Biomolecules and Biofluids

*Nicoló Riboni^1^, Alessandro Quaranta^1^, Hitesh V. Motwani^1^, Nicklas Österlund^2^, Astrid Gräslund^2^, Federica Bianchi^3^, Leopold L. Ilag^1^**

^1^Department of Environmental Science and Analytical Chemistry, Stockholm University, Stockholm, SE

^2^Department of Biochemistry and Biophysics, Stockholm University, Stockholm, SE

^3^Department of Chemistry, Life Sciences, and Environmental Sustainability, University of Parma, Parma, IT

## Corresponding Author

*E-mail: leopold.ilag@aces.su.se

Contents

[TABLES AND FIGURES 3](#_Toc3195719)

[Experimental parameters 3](#_Toc3195720)

[Over time disaggregation of Aβ 1-40 aggregates 4](#_Toc3195721)

[Solvent effect 5](#_Toc3195722)

[Heme MS/MS analysis 6](#_Toc3195723)

[Acidic glycans released from TFN 7](#_Toc3195724)

[Neutral glycans released from AAT 8](#_Toc3195725)

[Biofluids lipids identification 10](#_Toc3195726)

[Adduct formation on standard proteins 14](#_Toc3195727)

[REFERENCES 15](#_Toc3195728)

## TABLES AND FIGURES

### Experimental parameters

*Table S1. Instrumental parameters used for the different SAPSI applications*

|  | **Aβ 1-40 peptides** | **Intact proteins and biofluids** | **N-Glycans** |
| --- | --- | --- | --- |
| Clip Voltage (kV) | 3.5 | 4 | 4 |
| Cone Voltage (V) | 120 | 120 | 80 |
| Source Offset (V) | 80 | 80 | 80 |
| Source Temperature (ºC) | 70 | 70 | 90 |
| Cone gas (L/h) | 100 | 100 | 100 |
| Mass range (Da) | 100 - 5000 | 100 - 4000 | 100 - 4000 |
| Scan time (s) | 1 | 1 | 1 |
| Trap collision energy (V) | NA | 25 - 35 | 28 – 33 |
| SAPSI solvent composition | H_2_O : IPA (1:1)  HCOOH 1% v/v | H_2_O : IPA (1:1)  HCOOH 1% v/v | H_2_O : ACN (1:1)  HCOOH 0.1% v/v |
| SAPSI flow rate (µL) | 5 | 5 - 10 | 2 - 5 |

### Over time disaggregation of Aβ 1-40 aggregates

*Table S2. m/z values and related charges observed for the detected oligomeric states of Aβ peptide*

| **Charge** | **Olygomeric State** | | | | | | | |
| --- | --- | --- | --- | --- | --- | --- | --- | --- |
|  | 1 | 2 | 3 | 4 | 5 | 6 | 7 | 8 |
| 1 |  |  |  |  |  |  |  |  |
| 2 | 2165.93 |  |  |  |  |  |  |  |
| 3 | 1444.28 | 2887.62 |  |  |  |  |  |  |
| 4 | 1083.49 | 2165.99 | 3248.41 |  |  |  |  |  |
| 5 | 866.96 | 1732.93 | 2598.96 |  |  |  |  |  |
| 6 |  | 1444.37 | 2165.91 | 2887.64 |  |  |  |  |
| 7 |  | 1238.13 | 1856.69 | 2475.21 | 3093.80 |  |  |  |
| 8 |  |  | 1624.69 | 2165.92 | 2707.18 | 3248.40 |  |  |
| 9 |  |  |  | 1925.38 | 2406.55 | 2887.61 |  |  |
| 10 |  |  |  | 1732.98 | 2165.91 | 2598.92 | 3031,94 | 3150.02 |
| 11 |  |  |  |  |  | 2362.76 |  | 3464.94 |


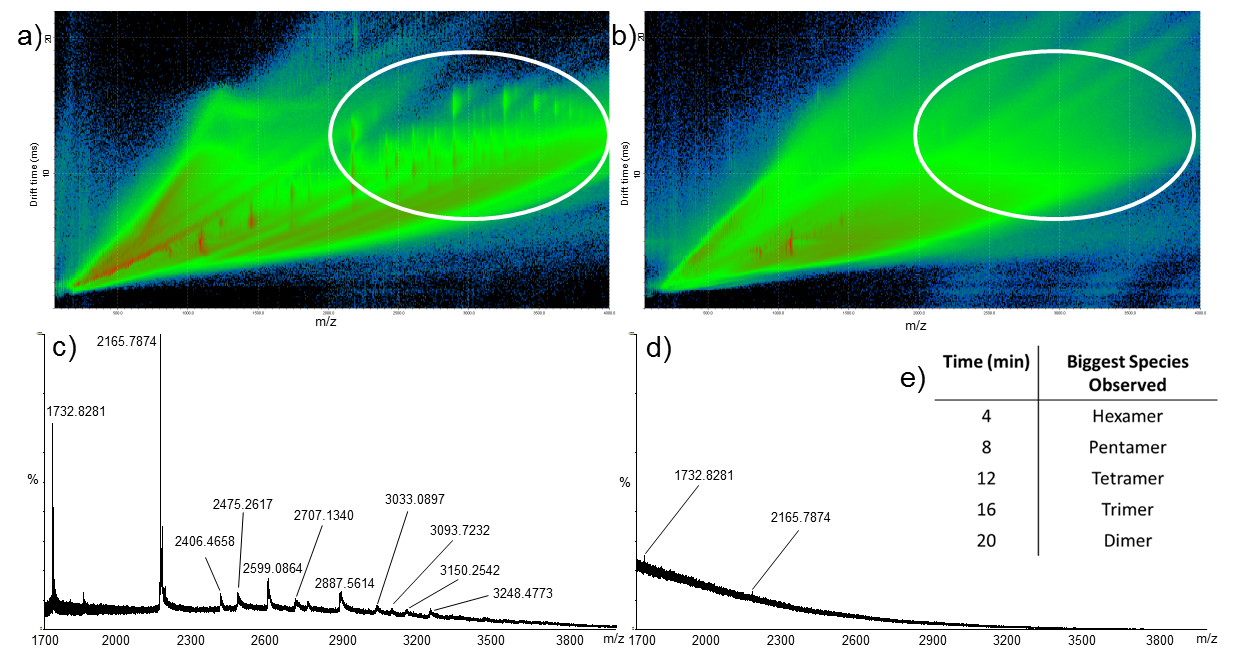


*Figure S1. Driftograms displaying the different drift times obtained for each m/z value for the real-time monitoring of the disaggregation of Aβ 1-40 aggregates over time, measured after 4 minutes (a) and 20 minutes (b). The region associated with m/z 1700 – 4000 is encircled. Corresponding MS spectra are reported in (c), 4 minutes, and (d), 20 minutes. The table (e) reports the largest aggregate species observed at each time point*

### Solvent effect

*Table S3. List of solvent mixtures (and their compositions) tested for the various SAPSI applications. Mixtures highlighted in bold text were found to perform best in the analysis of released neutral and acidic glycans*, intact proteins and biofluids**, and Aβ 1-40 monomer and aggregates****

| **Solvent** | **%** | **Additive** | **Additive concentration** |
| --- | --- | --- | --- |
| Water : MeOH | 50/50 | HCOOH | 1% v/v |
| Water : MeOH | 40/60 | HCOOH | 1% v/v |
| Water : MeOH | 35/60 | HCOOH | 5% v/v |
| Water : MeOH | 40/40 | HCOOH | 20% v/v |
| Water : MeOH | 40/20 | HCOOH | 40% v/v |
| Water : MeOH | 30/20 | HCOOH | 50% v/v |
| Water : MeOH | 40/20 | CH_3_COOH | 40% v/v |
| Water : MeOH | 30/20 | CH_3_COOH | 50% v/v |
| Water : ACN | 70/30 | HCOOH | 0.1% v/v |
| Water : ACN | 50/50 | HCOOH | 1% v/v |
| Water : ACN | 50/50 | CH_3_COOH | 1% v/v |
| **Water : ACN*** | **50/50** | **HCOOH** | **0.1% v/v** |
| Water : IPA | 90/10 | HCOOH | 5% |
| **Water : IPA**** | **50/50** | **HCOOH** | **1%** |
| **Water : IPA***** | **50/50** | **HCOOH** | **1%** |
| Tol : MeOH | 50/50 | HCOOH | 0.1% |


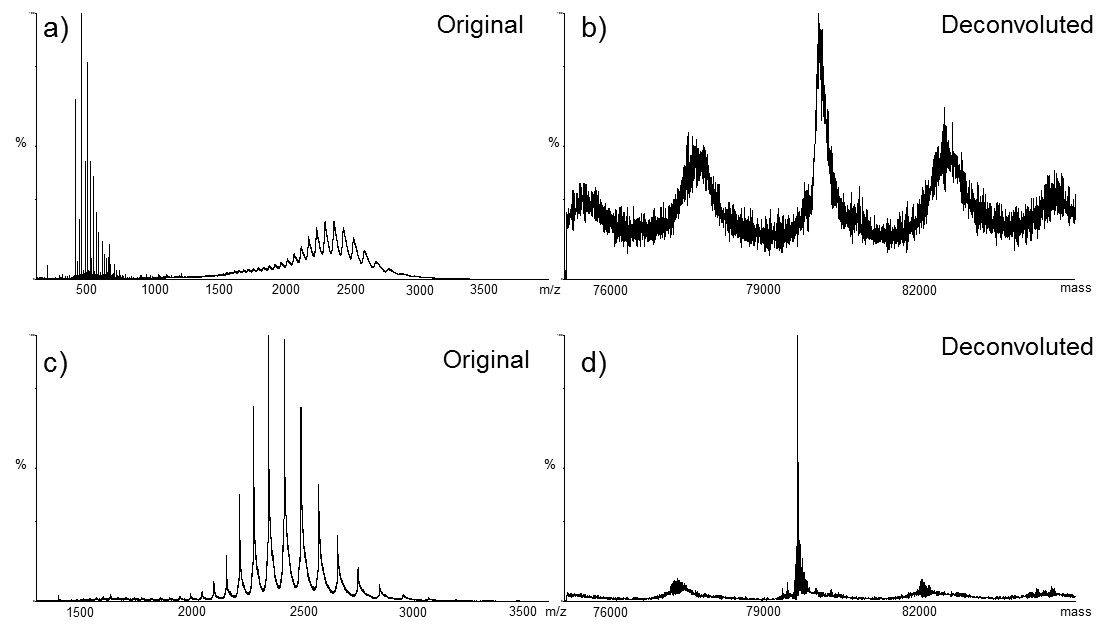


*Figure S2. MS spectrum of TFN with* *water:ACN = 1:1, HCOOH 1% v/v as solvent (a) and related MaxEnt™1 deconvolution in the range 75-85 kDa (b). MS spectrum of TFN with* *water:IPA = 1:1, HCOOH 1% v/v as solvent (c) and related MaxEnt™1 deconvolution in the range of 75-85 kDa (d)*

### Heme MS/MS analysis


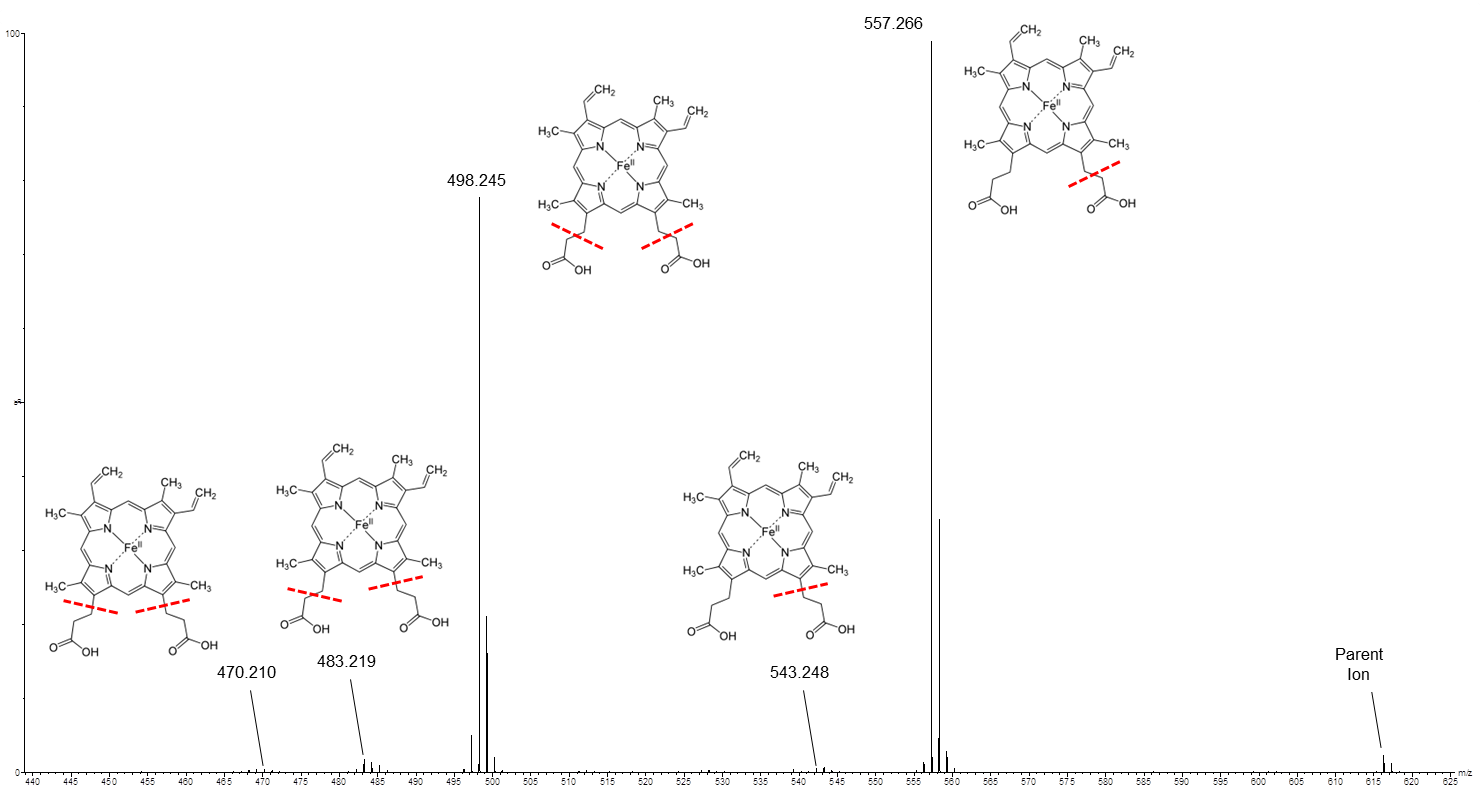


*Figure S3. MS/MS fragmentation of the heme group (m/z 616.2798) and assignment of fragments*

### Acidic glycans released from TFN


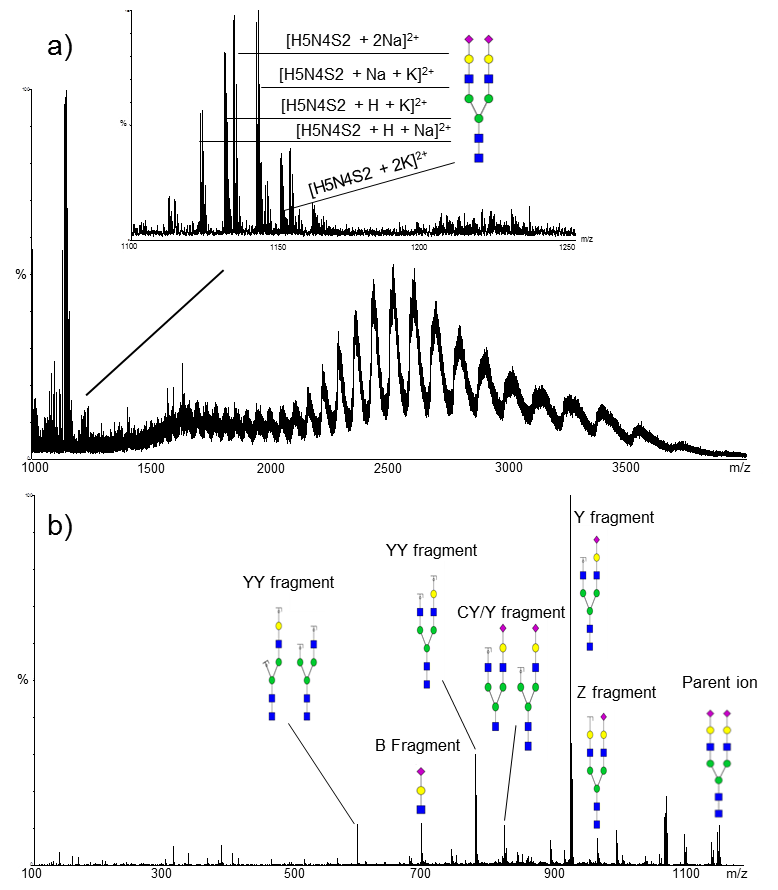


*Figure S4. MS spectrum from the analysis of acidic glycans released with PNGase F from TFN (a) and MS/MS fragmentation of m/z 1150.3110 and assignment of the fragments (b)*

### Neutral glycans released from AAT


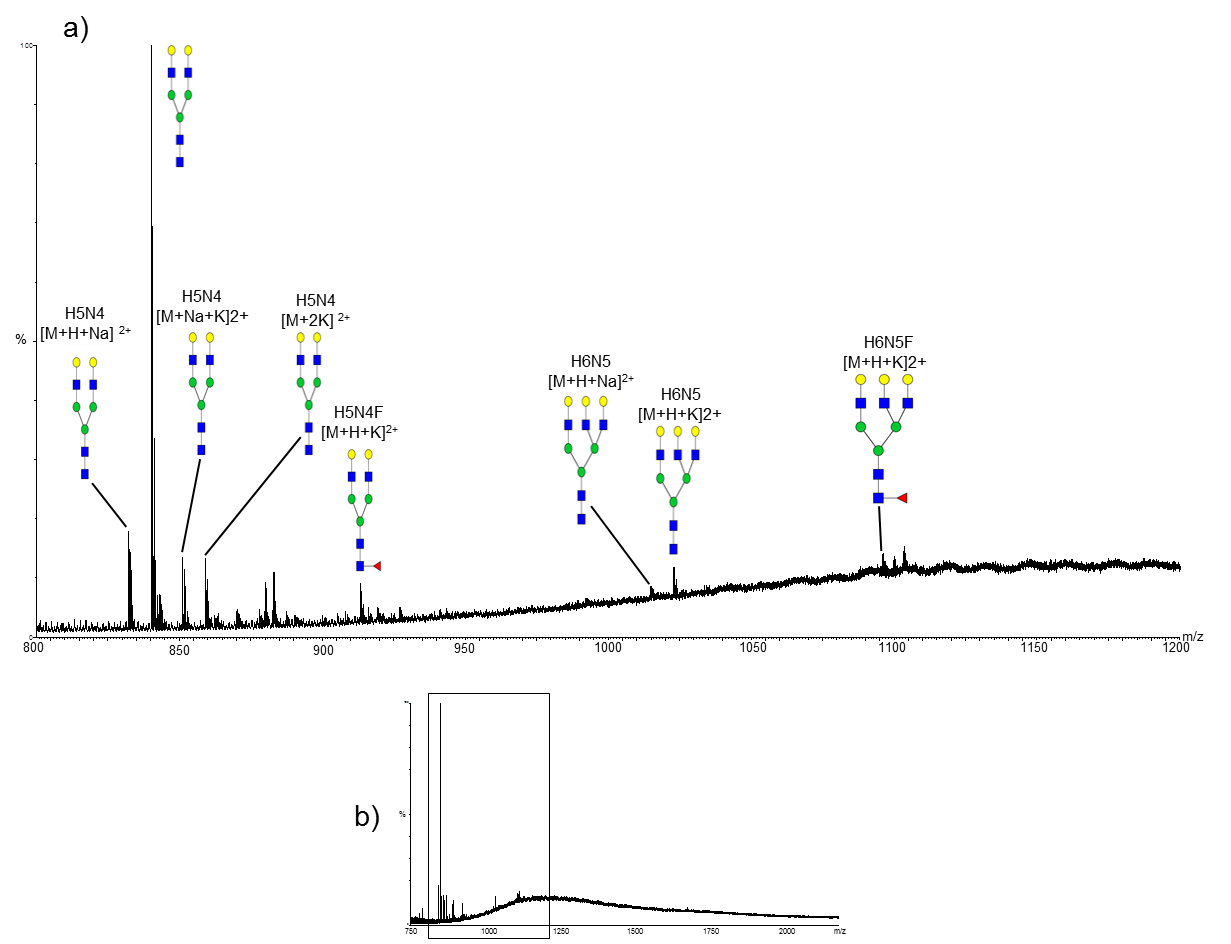


*Figure S5. Zoomed section of MS spectrum from the analysis of neutral glycans released with PNGase F from AAT (a) and full range MS spectrum with highlight on the glycan region (b)*


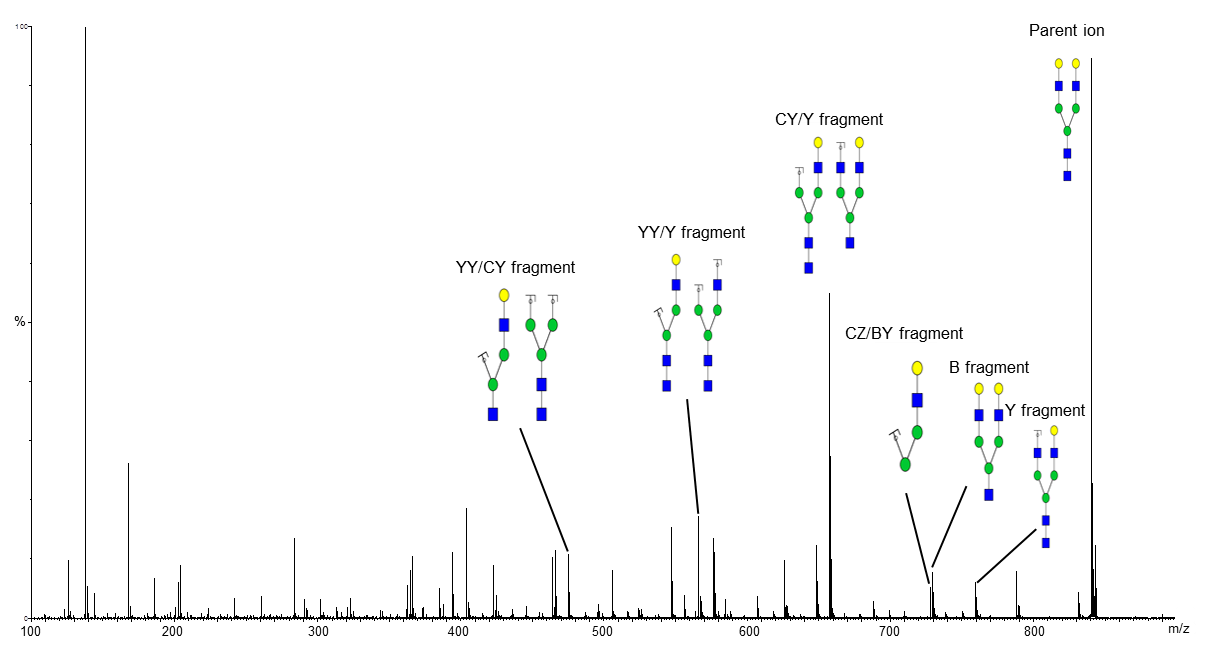


*Figure S6. MS/MS fragmentation of m/z 840.2404 and assignment of the fragments*

Cerebrospinal fluid analysis


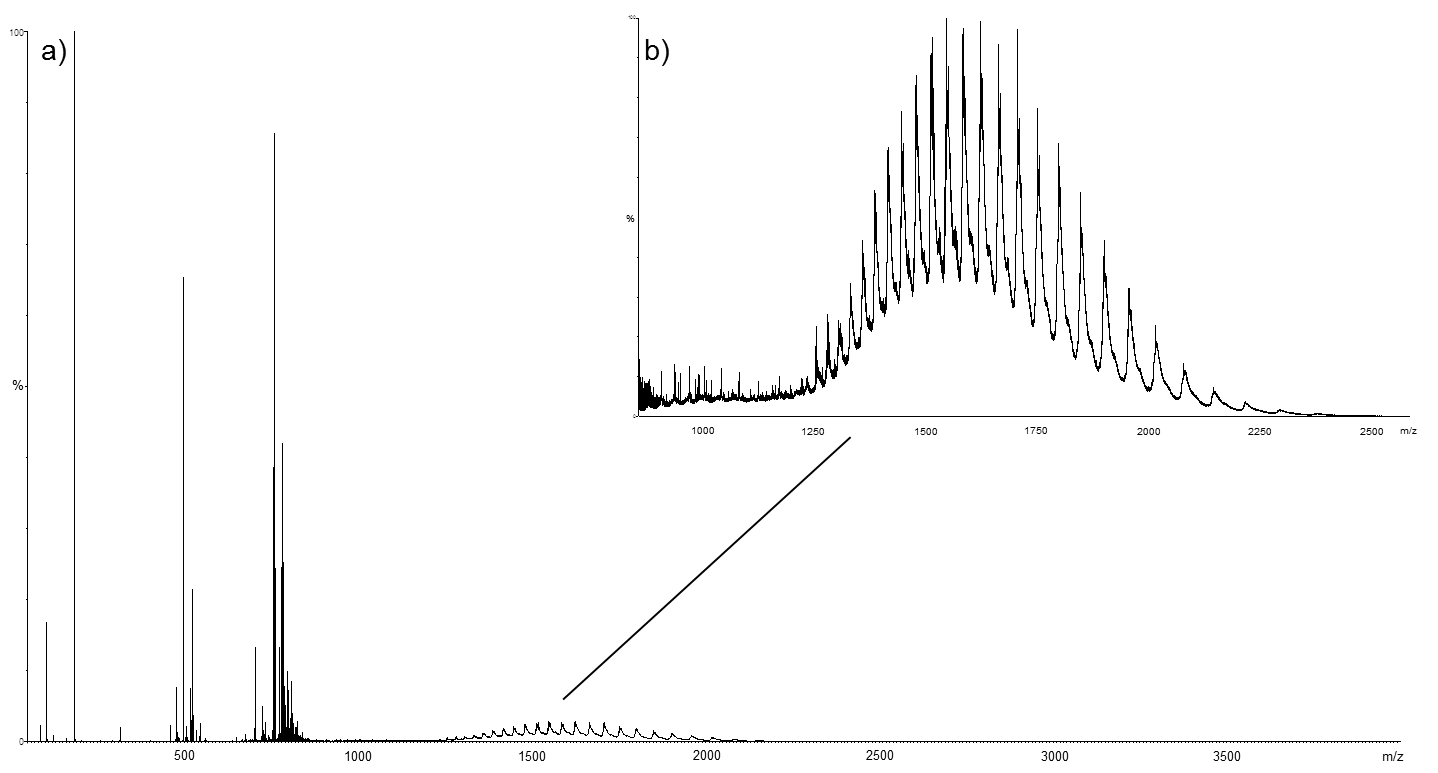


*Figure S7. MS spectrum acquired from a CSF sample, from the sum of 100 scans (a) and zoom in the m/z region of the HSA charge series envelope (b)*


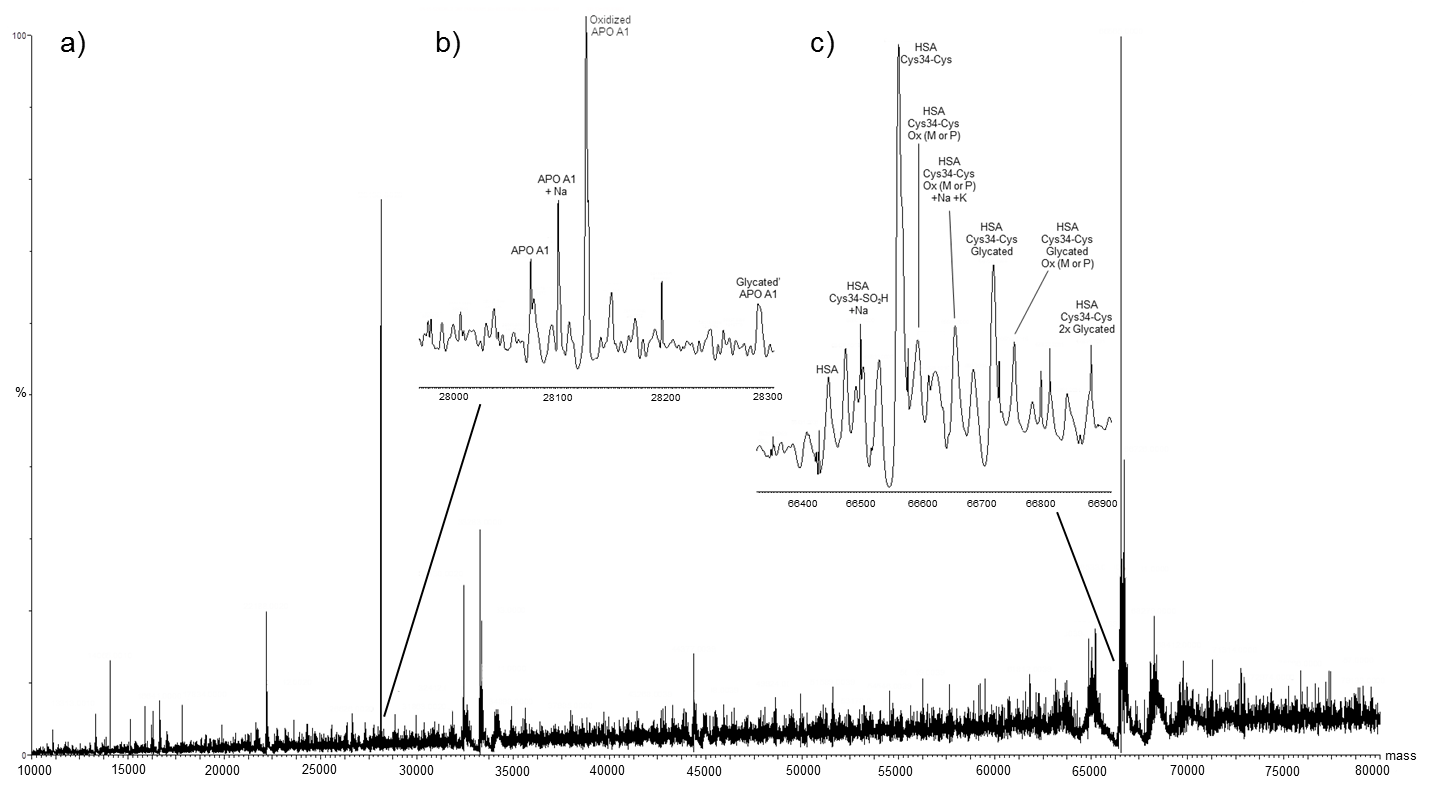


*Figure S8. MaxEnt™1 deconvolution of the spectrum depicted in Fig. S8 in the 10-80 kDa range (a). Zoom of the ApoA1 (b) and HSA (c) mass regions with identification of the different species.*

### Biofluids lipids identification


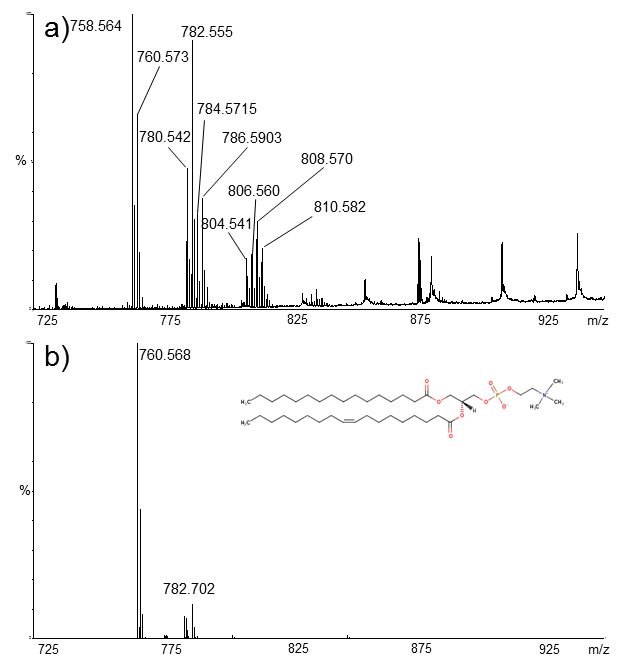


*Figure S9. MS spectrum of diluted serum sample expanded around the lipid region (a) compared with MS spectrum of standard PC 16:0-18:1 (b)*


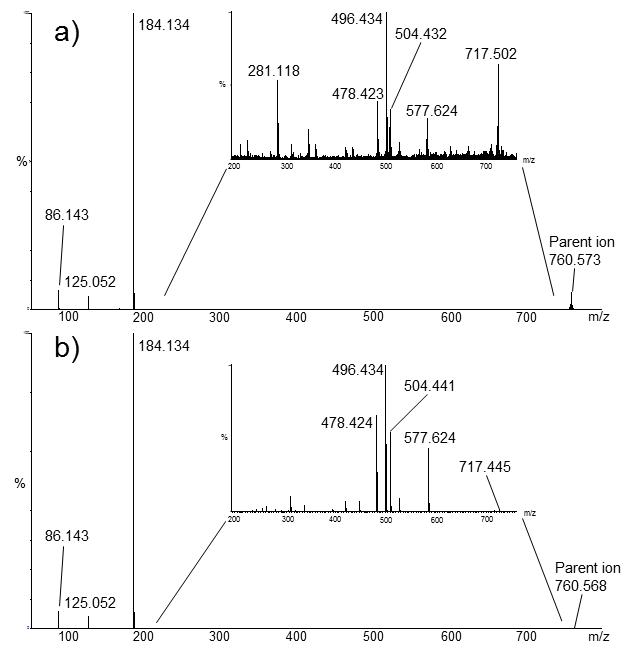


*Figure S10. MS/MS spectrum obtained from the fragmentation of m/z 760.573 species in diluted serum (a) compared to the MS/MS spectrum of standard PC 16:0-18:1 (m/z 760.568). For both spectra the regions in the range from 200 to 720 m/z were enlarged. It is possible to observe that the two obtained fragmentation patterns were comparable.*


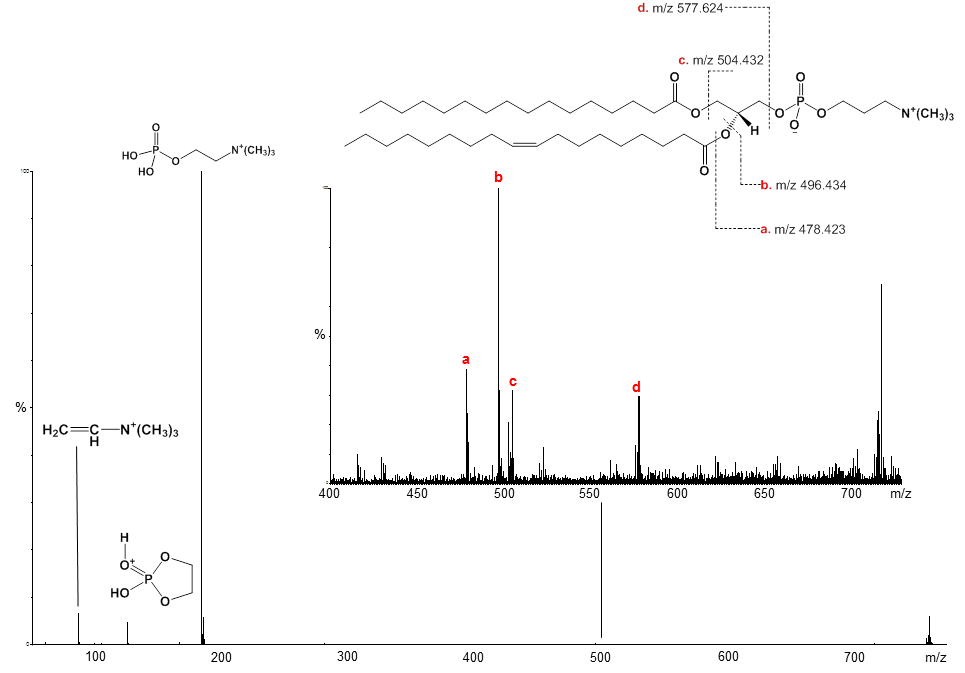


*Figure S11. Fragment assignment for PC 16:0-18:1. Fragmentation was consistent with literature data*[*^1^*](#_ENREF_1)


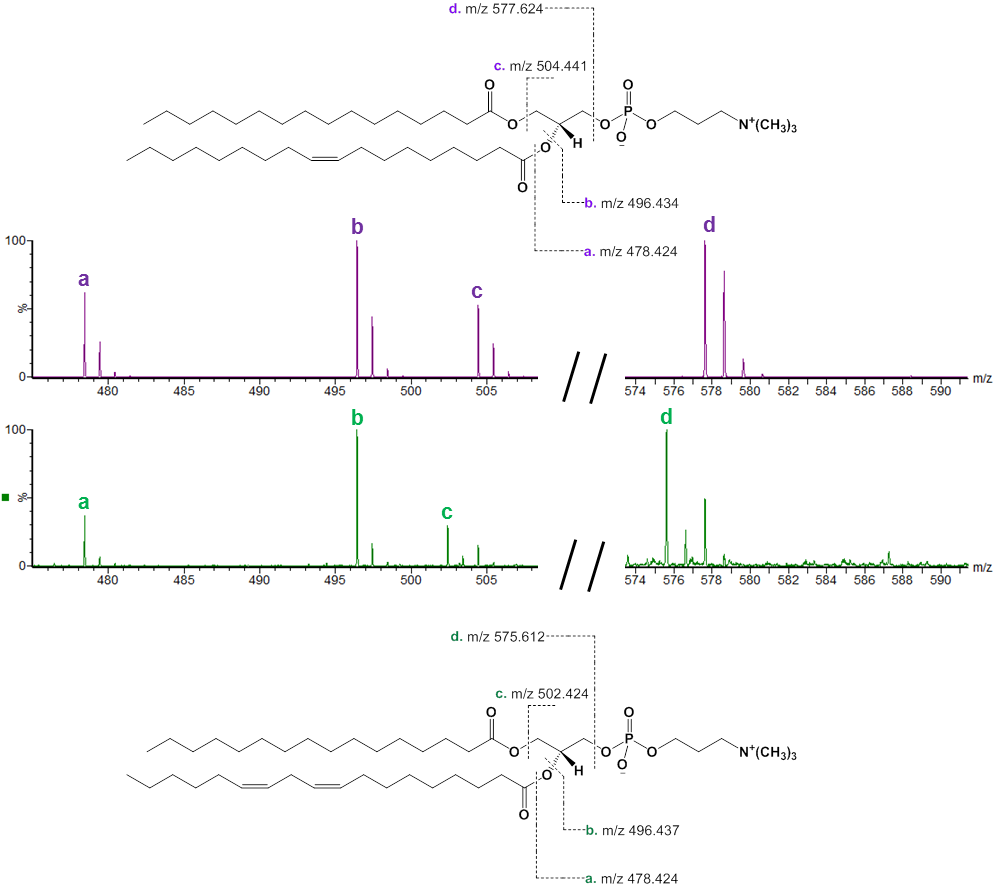


*Figure S12. Comparison between MS/MS fragmentation of m/z 760.568 in standard (purple) and m/z 758.564 in serum (green). While peaks a and b, related to the loss of the unsaturated acyl moiety, do not show a shift in mass, fragments c and d present a -2 Da mass difference. This indicates a further unsaturation on m/z 758.568, showing the presence of a linoleic acid unit instead of the oleic acid chain (PC 16:0-18:2).*

### Adduct formation on standard proteins


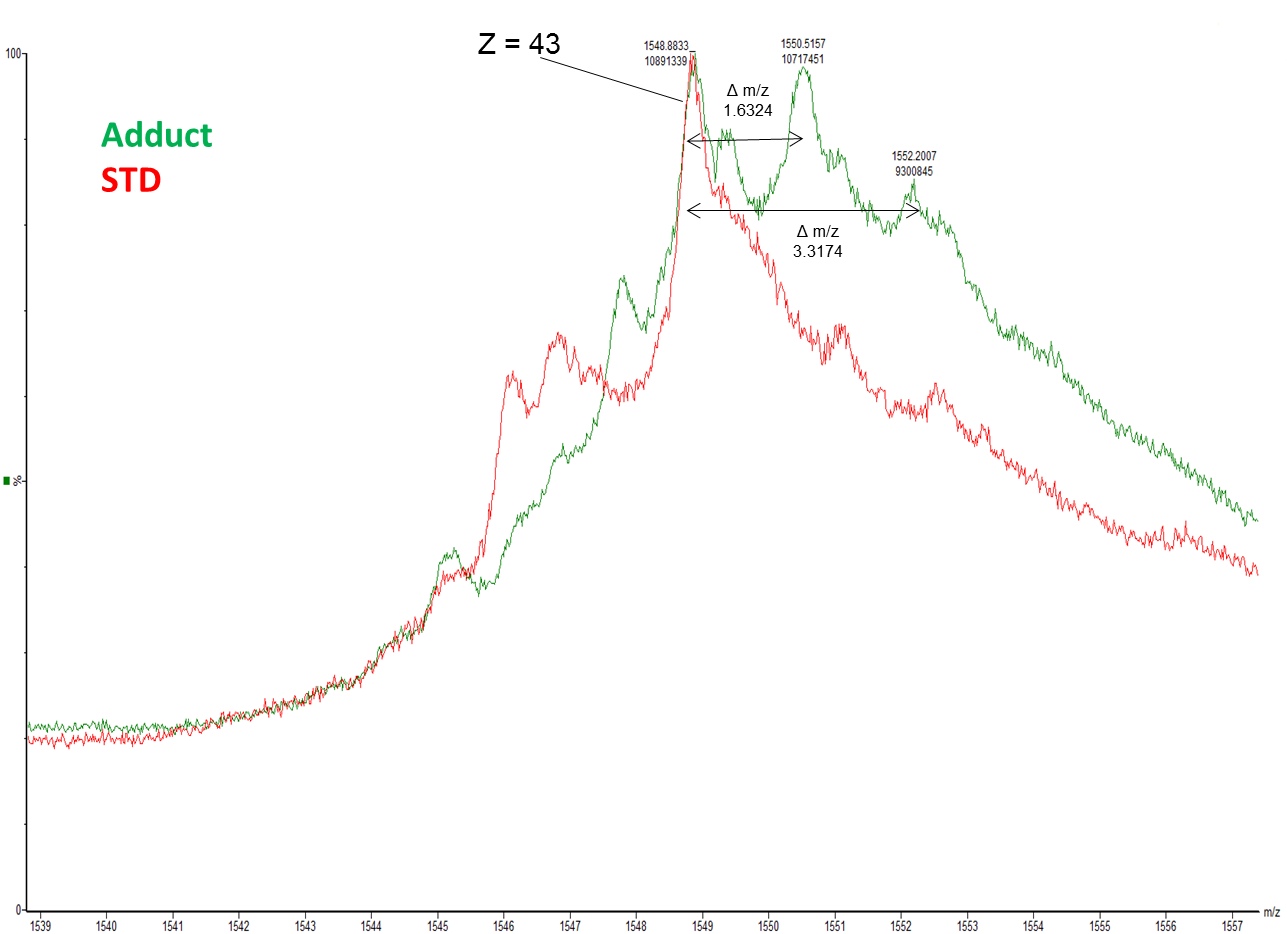


*Figure S13. MS overlapping of HSA +43 charge state in standard (red) or after alkylation with 30 µM AA (green). Different peaks, related to the mono-AA adduct and di-AA adduct with Δ 1.6324 and 3.3174 m/z respectively, were present in the spectrum of the alkylated sample*


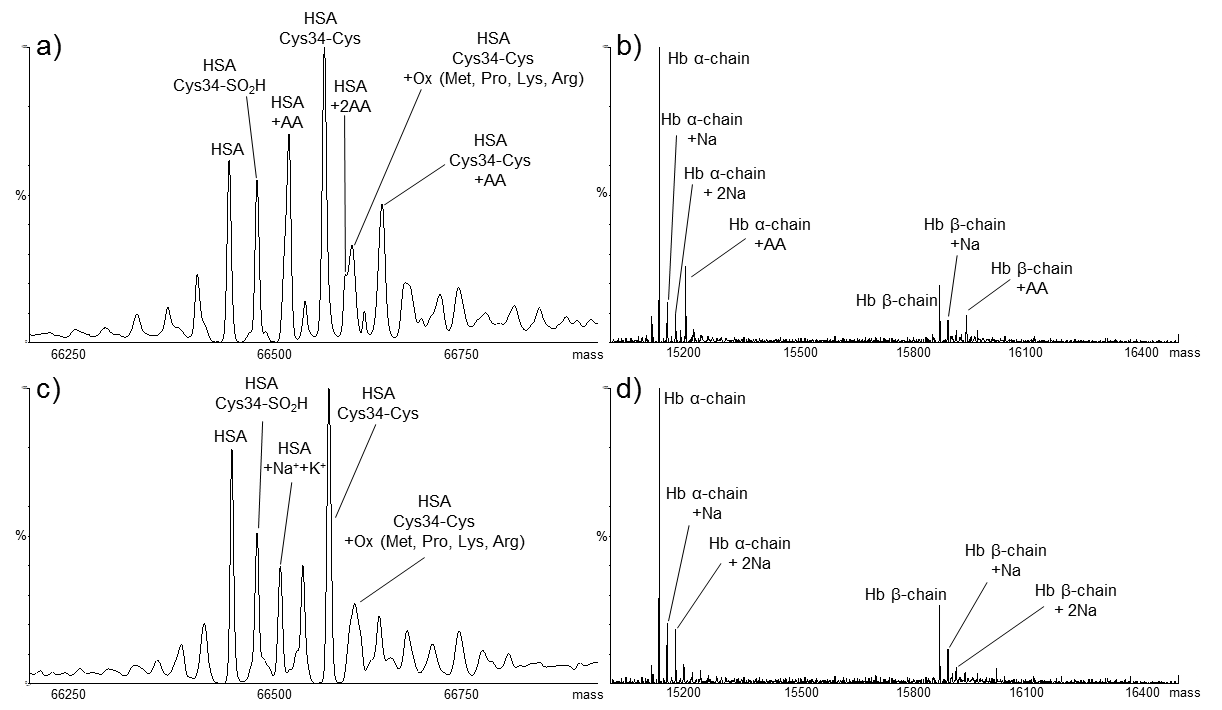


*Figure S14. MaxEnt™1 deconvolution of standard HSA and Hb after incubation with AA. HSA was incubated with AA 30 µM (a) and 0.3 µM (c), showing adducts only at the higher level. Hb was incubated with AA 30 µM (b) and 0.3 µM (d), showing adducts only at the higher level*

## REFERENCES

(1) Chughtai, K.; Jiang, L.; Greenwood, T. R.; Glunde, K.; Heeren, R. M. *Journal of lipid research* **2013**, *54*, 333-344.
